# Development of the Adult PedsQL™ Neurofibromatosis Type 1 Module: Initial Feasibility, Reliability and Validity

**DOI:** 10.1186/1477-7525-11-21

**Published:** 2013-02-21

**Authors:** Kavitha Nutakki, Cynthia M Hingtgen, Patrick Monahan, James W Varni, Nancy L Swigonski

**Affiliations:** 1 Department of Pediatrics, Indiana University School of Medicine, Indianapolis, IN, USA; 2 Department of Clinical Neurosciences, Spectrum Health Medical Group and College of Human Medicine, Michigan State University, Grand Rapids, MI, USA; 3 Department of Biostatistics, Indiana University School of Medicine, Indianapolis, IN, USA; 4 Department of Pediatrics, College of Medicine, Texas A&M University, College Station, TX, USA; 5 Department of Landscape Architecture and Urban Planning, College of Architecture, Texas A&M University, College Station, TX, USA; 6 Department of Public Health, Indiana University School of Medicine, Indianapolis, IN, USA

**Keywords:** PedsQL™, Neurofibromatosis type 1 (NF1), Health-related quality of life, Patient reported outcomes

## Abstract

**Background:**

Neurofibromatosis type 1 (NF1) is a common autosomal dominant genetic disorder with significant impact on health-related quality of life (HRQOL). Research in understanding the pathogenetic mechanisms of neurofibroma development has led to the use of new clinical trials for the treatment of NF1. One of the most important outcomes of a trial is improvement in quality of life, however, no condition specific HRQOL instrument for NF1 exists. The objective of this study was to develop an NF1 HRQOL instrument as a module of PedsQL™ and to test for its initial feasibility, internal consistency reliability and validity in adults with NF1.

**Methods:**

The NF1 specific HRQOL instrument was developed using a standard method of PedsQL™ module development – literature review, focus group/semi-structured interviews, cognitive interviews and experts’ review of initial draft, pilot testing and field testing. Field testing involved 134 adults with NF1. Feasibility was measured by the percentage of missing responses, internal consistency reliability was measured with Cronbach’s alpha and validity was measured by the known-groups method.

**Results:**

Feasibility, measured by the percentage of missing responses was 4.8% for all subscales on the adult version of the NF1-specific instrument. Internal consistency reliability for the Total Score (alpha =0.97) and subscale reliabilities ranging from 0.72 to 0.96 were acceptable for group comparisons. The PedsQL™ NF1 module distinguished between NF1 adults with excellent to very good, good, and fair to poor health status.

**Conclusions:**

The results demonstrate the initial feasibility, reliability and validity of the PedsQL™ NF1 module in adult patients. The PedsQL™ NF1 Module can be used to understand the multidimensional nature of NF1 on the HRQOL patients with this disorder.

## Background

Neurofibromatosis type 1 (NF1) is a common autosomal dominant genetic disorder with a prevalence of 1 in 3000 persons worldwide, independent of gender, race and ethnicity [1-4]. According to National Institutes of Health, NF1 is diagnosed by the presence of two or more of the following clinical features – 1) six or more Café-au-lait spots (>5 mm in prepubertal individuals and >15 mm in postpubertal individuals, both in greatest diameter), 2) two or more neurofibromas of any type or one plexiform neurofibroma, 3) freckling in the axillary or inguinal regions, 4) optic glioma, 5) two or more Lisch nodules, 6) a distinctive osseous lesion (sphenoid dysplasia or thinning of the long bone cortex with or without pseudarthrosis), 7) having a first degree relative with NF1 [5,6]. NF1, also known as von Recklinghausen’s disease, is characterized by the presence of multiple cutaneous neurofibromas, café au lait spots, intertriginous freckling, and Lisch nodules (iris hamartomas) [7]. Almost one half of the affected individuals with this disorder have been shown to have learning disabilities [8]. Other common findings include optic gliomas, bony abnormalities, headache and hypertension. Significant, but less common complications of NF1 include malignant peripheral nerve sheath tumors, brain tumors, vasculopathy, epilepsy, growth problems, neurological dysfunction and pruritus [1,7-9].

Neurofibromas, pathognomonic for NF1, are benign nerve sheath tumors that can be extraneural or intraneural. They are composed of Schwann cells, perineural cells, fibroblasts, and mast cells [10,11]. They may remain asymptomatic or can cause a wide variety of symptoms including pain, pruritus, paresthesias (tingling, numbness) and local trauma. Extraneural neurofibromas cause cosmetic disfigurement whereas internal neurofibromas impinge on neighboring organs and significantly increase morbidity and mortality [12,13]. Childhood through early adulthood is a critical period for the accelerated growth of neurofibromas [1,14]. Rapid tumor growth also occurs during pregnancy due to associated hormonal changes [12,14]. The variable nature of neurofibromas and other symptoms associated with NF1 have a significant impact on the health-related quality of life (HRQOL) of individuals with this disorder [15,16].

NF1 is a lifelong, progressive, variable and unpredictable disorder [17]. The main stay of treatment for NF1 is supportive or surgical. However, surgical removal of neurofibromas is unsatisfactory as these tumors often regrow and the underlying cause has not been treated [14,18]. Progress in understanding the genetics and pathogenetic mechanisms has led to the use of new drugs for the treatment of NF1 and the emergence of a number of clinical trials [11,19]. Participants in these clinical trials need objective follow up to monitor changes in clinical symptoms. Radiographic imaging (3-dimensional MRI) is being done with some success however, defining success from calculations of tumor mass from radiographic imaging is difficult as neurofibromas have irregular shapes and may be fibrotic [14]. Thus, they may not show a significant decrease in size with treatments despite the report of improvement in clinically significant symptoms [18]. Patients with little tumor shrinkage have anecdotally reported large improvements in functioning and well-being that could be measured with a NF1 specific HRQOL instrument.

HRQOL is arguably one of the most important measures in evaluating effectiveness of clinical treatments [20,21]. HRQOL instruments used in previous studies in patients with NF1 have been generic and may be useful for comparing across different health conditions. Studies of generic instruments showed that NF1 had a significant impact on all domains of the Short Form 36 health survey (SF-36) when compared to the normative population [15,16]. Limitations exist to generic quality of life survey instruments when they are applied to patients with specific illnesses [22]. Generic instruments do not measure disease-specific HRQOL, for instance, skin paresthesias in individuals with NF1. In contrast, disease-specific instruments measure the impact of specific symptoms and are more sensitive for the detection and quantification of small changes over time [22]. A significant gap in the current empirical literature is the lack of a validated NF1-specific HRQOL instrument. Consequently, the objective of this study was to develop an NF1 specific HRQOL instrument (as a Module of the PedsQL™) and to test for its initial feasibility, internal consistency reliability and validity. We hypothesized that HRQOL when measured by the PedsQL™ NF1 Module domains would be associated with the self-reported health status.

## Methods

Human subjects ethics for this study was reviewed and approved by the Indiana University Institutional Review Board and in compliance with the Helsinki Declaration. In accordance with HRQOL instrument development protocols, the PedsQL™ NF1 Module was designed through the following five phases – 1) literature review; 2) outline of the instrument; 3) pilot instrument development – a) focus group/semi-structured interviews, b) cognitive interviews, c) experts’ review; 4) pilot testing and 5) field testing.

### Phase 1 – literature review

We conducted an extensive literature search in Pub Med database for symptoms and signs of NF1 [5-9,23-34] (complete search methods and table of references available on request). The HRQOL literature was reviewed for measures pertinent to NF1 [35-39].

### Phase 2 – outline of the instrument

Clinicians taking care of patients with NF1 at Indiana University Hospitals, Indianapolis, IN, were interviewed to learn about their experiences with NF1. An initial outline of the instrument was developed based on literature review and clinicians’ experiences. Pertinent questions were drawn from the existing PedsQL™ Arthritis, Cancer, Cerebral Palsy and Family Impact Modules [36-39]. Instrument domains were designed to address HRQOL issues specific to NF1.

### Phase 3 – pilot instrument development

The initial instrument was modified after conducting a focus group or semi-structured interview, cognitive interviews and experts’ review. a) Focus group/Semi-structured interview: Information about the focus group was advertised in the NF clinic at Indiana University Hospitals. Individuals were enrolled into the focus group if they had NF1 and were willing to talk about their disorder and its effect on their health and well-being. Written-informed consent was obtained from all participants. We conducted one focus group of three individuals and 2 semi-structured interviews with two adults with NF1. Participants were encouraged to speak about how NF1 affected their health and well-being. All interviews were digitally recorded, transcribed and de-identified for research purposes. By interpretative phenomenological analysis [40,41], new domains were identified from the focus group/semi-structured interviews, specifically, Skin Irritation, Sensation, Movement and Balance and Sexual Functioning. b) Cognitive interviews: An initial draft instrument was administered at the NF clinic. Cognitive interviewing of the participants was done to find out problems with wording of items, interpretation of instructions and to estimate the time required to complete the surveys. The items were revised after receiving the participants’ feedback. c) Experts’ review: The modified instrument was further reviewed by NF1 researchers and clinicians. After cognitive interviews and expert reviews, additional changes were made which included rewording “sensitive skin” to “rough skin” in the Skin Irritation domain and adding “not applicable” as a choice in the Sexual Functioning domain. The pilot instrument developed as a result has 16 domains and 74 items.

The 74-item PedsQL™ NF1 Module: Adult self- report instrument comprises 16 domains/subscales: 1) Physical Functioning (8 items), 2) Emotional Functioning (5 items), 3) Social Functioning (3 items), 4) Cognitive Functioning (5 items), 5) Communication (3 items), 6) Worry (7 items), 7) Perceived Physical Appearance (3 items), 8) Pain and Hurt (3 items), 9) Paresthesias (2 items), 10) Skin Irritation (5 items), 11) Sensation (4 items), 12) Movement and Balance (4 items), 13) Daily Activities (12 items), 14) Fatigue (3 items), 15) Treatment Anxiety (4 items) and 16) Sexual Functioning (3 items).

The NF1 HRQOL format, instructions, and Likert response scale are similar to the PedsQL™ 4.0 Generic Core Scales and other PedsQL™ Disease-Specific Modules. Although originally developed for use in children, the PedsQL™ format has been extended to adults with a generic instrument as well as several disease specific instruments [35]. The instructions ask how much of a problem each item has been during the past one month. A 5-point response scale is used for all items (0 = never a problem, 1 = almost never a problem, 2 = sometimes a problem, 3 = often a problem, 4 = almost always a problem). Items are reverse scored and linearly transformed to a scale of 0–100 similar to PedsQL™ 4.0 Generic Core Scales (0 = 100, 1 = 75, 2 = 50, 3 = 25, 4 = 0) [35]. Hence, higher scores signify better HRQOL [42,43] and fewer symptoms or problems. The Total Score is computed as the sum of all items on the PedsQL™ NF1 Module divided by the number of items answered (this accounts for missing data). Subscale scores are computed as the sum of the items divided by the number of items that were answered in that subscale. If more than 50% of the items in the subscale are missing, the subscale score is not computed [44]. Information about demographics is not included in the instrument except for age of the participant. In addition, participants were asked to rate their health status on a Likert scale as – excellent, very good, good, fair, or poor.

### Phase 4 – pilot testing

The PedsQL™ NF1 pilot instrument was tested at the Children’s Tumor Foundation sponsored NF forum in July 2011 in Minnesota, to check for the initial feasibility and internal consistency reliability. All participants with NF1 who were not involved in prior phases of instrument development, were encouraged to fill out the surveys. A sample of 10 adults with NF1 completed the surveys. The mean age of the participants was 40.5 y (range 20 to 62 y). Feasibility was measured by the average time taken to complete the survey and by the percentage of missing responses [44]. Internal consistency reliability was measured by computing Cronbach’s coefficient alpha [45]. Pilot testing showed that, on an average, participants took 6 minutes to complete the survey (range 4 to 8 min). The missing response rate was 2.3% (n = 17), with the Sexual Functioning domain having the highest number of missing responses (n = 10). Two participants were missing 1 response in Physical Functioning, one participant was missing 3 responses in Perceived Physical Appearance, one participant was missing 1 response and three participants were missing all 3 responses in the Sexual Functioning domain. The Worry and Movement & Balance domains each had 1 missing response. Total scale internal consistency reliability was 0.82, showing adequate initial internal consistency of the instrument. Pilot testing provided initial support with adequate feasibility and internal consistency to proceed to the next phase of instrument development.

### Phase 5 – field testing

This final phase consisted of a larger NF1 sample across the country to determine feasibility, reliability and validity of PedsQL™ NF1 Module scales.

### Sample & setting

Participants were recruited from the NF clinic at Indiana University hospitals and national NF1 conferences from July 2011 to February 2012. In addition, the NF1 module for adults was placed online and web-links were advertised through NF organizations by publishing information about the study in their newsletters and websites (NF Midwest and Texas NF foundation). A sample of 124 adults completed the surveys. Pilot surveys (n = 10) are included in this sample since they showed no significant difference in mean subscale scores by independent samples t-test (*p* > 0.05). Mean age of the participants was 40.2 years, ranging from 20 to 71 years.

### Statistical analysis

Scale internal consistency reliability was determined by calculating Cronbach’s coefficient alpha [45]. For each subscale, “Cronbach’s alpha if item deleted” was determined to see if subscale reliability improves with the removal of the item. Subscale reliabilities of 0.70 or more are recommended for comparing patient groups, whereas reliability of 0.90 is recommended for analyzing individual patient scale scores [46,47]. Considering the small sample size, the Sexual Functioning domain was excluded from the analyses as it had the highest missing responses. Exploratory factor analysis using Promax rotation was conducted for the remaining 71 items. Item loadings were assessed using a cut-off value of 0.30 to see whether items loaded high on one and only one factor (i.e., ‘simple structure’) and whether the collection of items that loaded high on each factor formed a conceptually relevant subscale.

Feasibility was measured by the percentage of missing values [44]. Multitrait scaling analysis was performed to find out the extent to which individual items correlated with the hypothesized subscale construct rather than with other subscales [48]. We also examined the item-hypothesized subscale correlations (corrected by removing the item from the Total Score) and we used a cutoff of 0.40 or higher for indicating good item discrimination [44,45]. Multitrait scaling analyses were summarized via tests of individual item scaling success, defined as the number of times an item correlated higher with its hypothesized subscale construct rather than with another subscale by ≥ 2 standard errors [44], which provided an approximation of scaling success. The percentage of item scaling successes relative to the total number of item scaling tests was calculated for each subscale [44,49].

Construct validity of the instrument was determined using the known-groups method, which compares subscale scores across groups known to differ in the health construct being investigated [44,50]. NF1 participants were divided into 3 groups based on their self-reported health status – ‘excellent to very good’ (n = 47), ‘good’ (n = 46), and ‘fair to poor’ (n = 41). Mean subscale scores were compared among these 3 groups using one-way ANOVA. Effect sizes were calculated for the subscale scores to estimate the magnitude of differences. Effect sizes are designated as small (.20), medium (.50) and large (.80) in magnitude [51]. Statistical analyses were done using SPSS 18 version for Windows (SPSS Inc., Chicago IL, USA) and SAS 9.3 version for Windows (SAS Institute Inc., Cary, NC, USA).

## Results

### Item reduction

Based on the combined results of item-test statistics while determining reliability and exploratory factor analysis, 4 items were deleted from the following subscales of the instrument – “having headaches (Physical Functioning), feeling isolated from others (Social Functioning), worry about keeping or doing a job (Worry) and managing my NF1 (Treatment Anxiety). The final instrument reported in this manuscript has 16 subscales and 70 items (including the Sexual Functioning domain) as shown in Appendix A.

### Feasibility

Feasibility, measured by the percentage of missing responses was 4.8% for all subscales on the adult version of the PedsQL™ NF1 Module. The Sexual Functioning subscale had the highest number of missing responses (124, 30.8%) and was not included in any statistical analyses.

### Item-internal consistency

Item-subscale correlations showed that all items on the adult version of the PedsQL™ NF1 Module exceeded our criterion (0.40) for item discrimination, except for one item on the Worry subscale (worry about future or the risk of having children with NF1) with a correlation of 0.34. We retained this item in the final version of the PedsQL™ NF1 Module, however, because it was deemed important by the NF1 adults during the focus group/semi-structured interviews and by NF1 experts.

### Item scaling tests

The results of scaling tests for the adult version of the PedsQL™ NF1 Module are shown in Table 1. The scaling success for Cognitive Functioning was highest with 100% and lowest for Social Functioning at 42.86%. The mean and median of scaling success for all the subscales of the adult version of the PedsQL™ NF1 Module was 73% and 71.4% respectively.

**Table 1:** **Item scaling tests for the adult PedsQL™ **NF1 module subscalesª

**Subscale**	**Items**^**b**^	**Range of item correlations**		**Item scaling tests**	
		**Item-Internal consistency**^**c**^	**Item-Discriminant validity**^**d**^	**# Success/Total**^**e**^	**Scaling success rate (%)**
Physical Functioning	7	.69-.85	.01 -.67	72/98	73.47
Emotional Functioning	5	.69-.85	.18 -.52	64/70	91.43
Social Functioning	2	.58	.14 -.47	12/28	42.86
Cognitive Functioning	5	.77-.90	.08-.46	70/70	100
Communication	3	.58-.81	.12 -.49	38/42	90.48
Worry	6	.34-.79	.01 -.56	51/84	60.71
Perceived Physical Appearance	3	.72-.85	.02 -.55	38/42	90.48
Pain and Hurt	3	.80-.93	.15 -.76	25/42	59.52
Paresthesias	2	.77	.16 -.72	16/28	57.14
Skin Irritation	5	.48-.76	.08 -.53	39/70	55.71
Sensation	4	.43-.64	.04 -.46	39/56	69.64
Movement and Balance	4	.74-.84	.08 -.69	38/56	67.86
Daily Activities	12	.59-.89	.01 -.65	140/168	83.33
Fatigue	3	.78-.83	.21-.60	30/42	71.43
Treatment Anxiety	3	.55-.86	.11 -.45	34/42	80.95

^a^ n = 134, Standard error = 0.09.

^b^ Number of items and number of item-internal consistency tests per subscale.

^c^ Correlations between items and hypothesized subscale corrected for overlap.

^d^ Correlations between items and other subscales.

^e^ Number of hypothesized significantly higher/total number of correlations.

### Internal consistency reliability

Table 2 shows internal consistency reliability coefficients for all subscales of the Adult PedsQL™ NF1 Module. Subscale reliabilities ranged from 0.72 to 0.96, with all subscales exceeding the minimum reliability criterion of 0.70 required for group comparisons. Total Score was 0.97, which exceeded the reliability criterion of 0.90 recommended for analyzing individual patient scores.

**Table 2:** **Reliability and descriptive statistics for the adult PedsQL™** NF1 module

**Domain**	**Items**	**Sample**	**Mean ± SD**	**α**
Physical Functioning	7	133	63.21 ± 28.75	.93
Emotional Functioning	5	134	51.53 ± 25.10	.92
Social Functioning	2	134	60.91 ± 27.40	.73
Cognitive Functioning	5	134	52.42 ± 25.14	.94
Communication	3	127	66.21 ± 27.38	.84
Worry	6	127	42.66 ± 24.83	.83
Perceived Physical Appearance	3	126	38.96 ± 33.41	.90
Pain and Hurt	3	127	53.61 ± 33.03	.93
Paresthesias	2	127	58.86 ± 31.38	.87
Skin Irritation	5	127	65.39 ± 25.03	.83
Sensation	4	124	67.14 ± 24.16	.72
Movement and Balance	4	125	72.60 ± 27.88	.91
Daily Activities	12	124	89.38 ± 20.09	.96
Fatigue	3	124	51.75 ± 28.42	.90
Treatment Anxiety	3	124	75.20 ± 25.95	.87
Total Score	67	134	63.07 ± 17.86	.97

Higher values equal better health-related quality of life. SD, standard deviation; α, Cronbach’s coefficient alpha.

### Construct validity

Table 3 compares mean subscale scores and effect sizes of three groups of NF1 participants based on their self-reported health status. Total Scores of the three groups showed statistically significant differences, with a lower score among NF1 participants with ‘fair to poor’ health status. All subscale scores of the instrument were significantly different among the three groups, supporting initial discriminant validity of the PedsQL™ NF1 Module. Effect sizes ranged from 0.22 to 0.63, with the largest effect sizes for the Pain and Hurt subscale, and the lowest effect sizes for the Perceived Physical Appearance subscale. The majority of the effect sizes were in medium range supporting discriminant validity of the individual subscales.

**Table 3:** Comparison of mean subscale scores among 3 groups of NF1 participants based on self-reported health status

**Subscale**	**Excellent - Very good**		**Good**		**Fair - Poor**		p-value*	**Effect size**
	**N**	**Mean ± SD**	**N**	**Mean ± SD**	**N**	**Mean ± SD**		
Physical Functioning	46	79.66 ± 24.79	46	64.51 ± 25.99	41	43.29 ± 23.61	<.0001	.51
Emotional Functioning^**#**^	47	60.64 ± 21.86	46	52.17 ± 26.32	41	40.37 ± 23.25	.001	.33
Social Functioning^**#**^	47	72.34 ± 26.57	46	59.78 ± 28.86	41	49.09 ± 21.17	<.0001	.35
Cognitive Functioning^**#**^	47	58.40 ± 26.66	46	54.02 ± 24.35	41	43.75 ± 22.25	.020	.24
Communication^**#**^	46	74.28 ± 26.11	43	65.50 ± 26.39	38	57.24 ± 27.75	.016	.25
Worry	46	51.38 ± 24.77	43	44.77 ± 24.65	38	29.71 ± 19.79	<.0001	.36
Perceived Physical Appearance^**#**^	46	46.92 ± 34.26	42	39.48 ± 35.69	38	28.73 ± 27.31	.044	.22
Pain and Hurt	46	74.09 ± 28.45	43	58.53 ± 25.10	38	23.25 ± 22.77	<.0001	.63
Paresthesias	46	73.10 ± 30.61	43	65.41 ± 25.71	38	34.21 ± 23.19	<.0001	.53
Skin Irritation	46	77.83 ± 20.46	43	65.23 ± 22.47	38	50.53 ± 25.14	<.0001	.44
Sensation^**#**^	45	74.03 ± 21.48	42	67.26 ± 22.51	37	58.61 ± 26.82	.015	.26
Movement and Balance	46	87.91 ± 20.04	42	74.26 ± 21.08	37	51.69 ± 30.29	<.0001	.53
Daily Activities	46	95.61 ± 15.45	42	94.49 ± 16.13	36	75.46 ± 22.91	<.0001	.45
Fatigue	46	63.04 ± 27.81	42	58.93 ± 24.72	36	28.94 ± 19.05	<.0001	.52
Treatment Anxiety	46	82.79 ± 23.33	42	77.78 ± 23.47	36	62.50 ± 27.78	.001	.32
Total Score	47	74.37 ± 14.03	46	64.63 ± 15.64	41	48.37 ± 13.60	<.0001	.59

*p-values based on one-way ANOVA. Higher values equal better health-related quality of life. Effect sizes are designated as small (0.20), medium (0.50) and large (0.80). SD, standard deviation. ^#^ Except for these, Fair-Poor health status group was different from other two groups with p < .05 by Bonferroni test.

## Discussion

The present study provides support for the initial feasibility, reliability and validity of the PedsQL™ NF1 Adult Version in a general population of adults with NF1. The adult version of the PedsQL™ NF1 Module could be completed in 6 minutes and demonstrated minimal missing values, supporting the feasibility of the instrument. The majority of the missing responses were shown for the Sexual Functioning subscale, which was not included in statistical analyses. We included this domain in the instrument in the appendix since it was reflective of concerns expressed by the majority of patients during the focus group/semi-structured interviews and may be an important area of improvement with newer therapies. Internal consistency for the Adult PedsQL™ NF1 Module Total Score exceeded the minimum reliability criterion of 0.90 for individual patient analysis, which supports the use of a Total Score as a measure of HRQOL in NF1 adults and supports the use of this instrument to follow improvement or deterioration over time in individuals. The individual subscale scores ranged from .72 to .96, which suggest that each subscale can be used to examine the specific domains of the PedsQL™ NF1 Module as well as using the Total Score for an overall assessment of NF1-specific HRQOL.

The adult version of PedsQL™ NF1 Module was able to differentiate among patients with varying overall health status. These findings support the initial discriminant validity of the Adult PedsQL™ NF1 Module. Consistent with our hypothesis, lower scores on the Adult PedsQL™ NF1 Module domains were associated with adult patients’ self-reported ‘fair’ to ‘poor’ health status. Adult patients with ‘excellent’ to ‘very good’ health status had higher HRQOL scores when compared to the other two health status groups across all subscales. The study sample has a mixture of participants from both clinical populations and general NF1 populations. The greatest difference in mean subscale scores existed in the Pain and Hurt subscale (50.84), which demonstrates that a clinic population reports more pain compared to rest of the participants. The Cognitive Functioning subscale (14.65) showed minimal differences among the groups. Although cognitive impairment is a frequent finding in NF1 [52], it is likely that the clinic group is presenting for physical symptoms.

Our study has several strengths, including the diversity of sample, nation-wide representation of the participants (clinic populations from states in and around Indiana, and a general NF1 population by advertising the study at national conferences and organizations) and broad age range (20-71 years) of participants in the field test. The Adult PedsQL™ NF1 Module can be self-administered, read easily (designed at the sixth grade reading level) and filled out quickly. Although the Module appears lengthy with 16 domains and 70 items, participants took an average of 6 minutes to complete it in pilot testing.

Our study also has some limitations. First of all, the sample size was somewhat small for a field test, which limits the precision of our factor analysis when reducing the number of items. Secondly, for divergent validity, we used participants self-reported health status and hence, there exists the possibility of overestimating or underestimating the actual disease severity. The Social Functioning subscale in the Adult PedsQL™ NF1 Module was problematic with a low scaling success. Although, we originally had 3 items in this subscale, one item was dropped to improve the subscale internal consistency. In future versions of this instrument we recommend testing more Social Functioning items as well as the Sexual Functioning items.

Currently, we are developing teen report and parent proxy report versions of the NF1 instrument. In the future, we plan to follow the strict methodology for PedsQL™ instrument development to validate child, teen and parent versions of the instrument.

## Conclusions

In summary, the adult version of PedsQL™ NF1 Module can be used to understand the multidimensional nature of NF1 on the HRQOL patients with this disorder and may assist in medical decision making. The instrument demonstrates initial feasibility, reliability, and discriminant validity.

## Appendix A

### PedsQL™ NF1 Module-Adult report

Physical Functioning

1. Feeling physically weak

2. Walking more than one block

3. Climbing stairs

4. Running

5. Doing a sports activity or exercise

6. Lifting something heavy

7. Doing chores around the house

Emotional Functioning

1. Feeling anxious

2. Feeling sad

3. Feeling angry

4. Feeling frustrated

5. Feeling helpless or hopeless

Social Functioning

1. Getting support from others

2. Having enough energy for social activities

Cognitive Functioning

1. Keeping attention on things

2. Remembering what people tell you

3. Remembering what you just heard/read

4. Thinking quickly

5. Remembering what you were just thinking

Communication

1. Telling the doctors and nurses how you feel

2. Asking the doctors and nurses questions

3. Talking with others about your disorder

Worry

1. Worrying about my neurofibromas

2. Worrying about side effects from medical treatments

3. Worrying about whether or not medical treatments are working

4. Worrying that neurofibromas will grow bigger or reoccur

5. Worrying about my future or the risk of having children with Neurofibromatosis type 1

6. Worrying about the risk of other health related issues associated with Neurofibromatosis type 1

Perceived Physical Appearance

1. Feeling that I am not good looking

2. Not wanting other people to see my neurofibromas

3. Being embarrassed about others seeing my body

Pain and Hurt

1. Aching or hurting

2. Aching or hurting a lot

3. Not sleeping because of pain

Paresthesias

1. A burning sensation in some part of my body

2. A tingling sensation in some part of my body

Skin Irritation

1. Itching

2. Itching a lot

3. Getting a skin rash when exposed to sun

4. Tolerating temperature changes

5. Rough skin

Sensation

1. Vision in one or both eyes

2. Seeing well enough with glasses or contact lenses

3. Hearing in one or both ears

4. Speech

Movement and Balance

1. Bending my body

2. Moving one or both legs

3. Using or moving one or both arms

4. Keeping balance when sitting or standing

Daily Activities

1. Putting on shoes

2. Buttoning my shirt

3. Combing my hair

4. Getting into the bathroom to use the toilet

5. Undressing to use the toilet

6. Getting in and out of bathtub or shower

7. Brushing my teeth

8. Eating with a fork and knife

9. Using a phone

10. Shopping

11. Managing money

12. Driving

Fatigue

1. Feeling tired

2. Resting a lot

3. Having enough energy to do things that I like to do

Treatment Anxiety

1. Getting scared about going to the doctor

2. Getting scared about going to the hospital

3. Being responsible for my medicines or therapy

Sexual Functioning

1. Fatigue or lack of energy affecting your satisfaction with your sex life

2. Pain affecting your satisfaction with your sex life

3. Ability to have children with a fertile partner

Copyright © 2012.

The PedsQL™ is available at http://www.pedsql.org/index.html.

## Abbreviations

PedsQL™: Pediatric quality of life inventor y™; NF1: Neurofibromatosis type 1; HRQOL: Health-related quality of life; ANOVA: Analysis of variance; SPSS: Statistical package for the social sciences; SAS: Statistical analysis software

## Competing interests

Dr. Varni holds the copyright and the trademark for the PedsQL™ and receives financial compensation from the Mapi Research Trust, which is a non-profit research institute that charges distribution fees to for-profit companies that use the Pediatric Quality of Life Inventory™.

## Authors’ contributions

NLS served as the Principal Investigator, conceptualized the rationale and designed the study. KN and NLS performed the literature review. KN, CMH and NLS worked for the data collection. KN, PM, JWV and NLS participated in statistical analysis. KN drafted the manuscript. NLS, PM, CMH and JWV provided critical review on all parts of the manuscript. All authors read and approved the final version of the manuscript.

## References

[B1] Lu-Emerson C Plotkin SR The neurofibromatoses. Part 1: NF1 Rev Neurol Dis 2009 6 E47 E53 19587630

[B2] Rasmussen SA Friedman JM NF1 gene and neurofibromatosis 1 Am J Epidemiol 2000 151 33 40 10.1093/oxfordjournals.aje.a010118 10625171

[B3] Lammert M Friedman JM Kluwe L Mautner VF Prevalence of neurofibromatosis 1 in German children at elementary school enrollment Arch Dermatol 2005 141 71 74 10.1001/archderm.141.1.71 15655144

[B4] Williams VC Lucas J Babcock MA Gutmann DH Korf B Maria BL Neurofibromatosis type 1 revisited Pediatrics 2009 123 124 133 10.1542/peds.2007-3204 19117870

[B5] Gutmann DH Aylsworth A Carey JC Korf B Marks J Pyeritz RE Rubenstein A Viskochil D The diagnostic evaluation and multidisciplinary management of neurofibromatosis 1 and neurofibromatosis 2 JAMA 1997 278 51 57 10.1001/jama.1997.03550010065042 9207339

[B6] Friedman JM Neurofibromatosis 1: clinical manifestations and diagnostic criteria J Child Neurol 2002 17 548 554 discussion 571–542, 646–551 10.1177/088307380201700802 12403552

[B7] Friedman JM Pagon RA, Bird TD, Dolan CR, Stephens K, Adam MP **Neurofibromatosis 1** GeneReviews 1993 Seattle (WA):

[B8] Jett K Friedman JM Clinical and genetic aspects of neurofibromatosis 1 Genet Med 2010 12 1 11 20027112 10.1097/GIM.0b013e3181bf15e3

[B9] North KN Neurofibromatosis 1 in childhood Semin Pediatr Neurol 1998 5 231 242 10.1016/S1071-9091(98)80002-8 9874851

[B10] Staser K Yang FC Clapp DW Mast cells and the neurofibroma microenvironment Blood 2010 116 157 164 10.1182/blood-2009-09-242875 20233971 PMC2910605

[B11] Yang FC Ingram DA Chen S Zhu Y Yuan J Li X Yang X Knowles S Horn W Li Y Nf1-dependent tumors require a microenvironment containing Nf1+/−− and c-kit-dependent bone marrow Cell 2008 135 437 448 10.1016/j.cell.2008.08.041 18984156 PMC2788814

[B12] Ablon J Gender response to neurofibromatosis 1 Soc Sci Med 1996 42 99 109 10.1016/0277-9536(95)00076-3 8745111

[B13] Sbidian E Wolkenstein P Valeyrie-Allanore L Rodriguez D Hadj-Rabia S Ferkal S Lacour JP Leonard JC Taillandier L Sportich S NF-1Score: a prediction score for internal neurofibromas in neurofibromatosis-1 J Invest Dermatol 2010 130 2173 2178 10.1038/jid.2010.100 20428190

[B14] Rosser T Packer RJ Neurofibromas in children with neurofibromatosis 1 J Child Neurol 2002 17 585 591 discussion 602–584, 646–551 10.1177/088307380201700808 12403557

[B15] Wolkenstein P Zeller J Revuz J Ecosse E Leplege A Quality-of-life impairment in neurofibromatosis type 1: a cross-sectional study of 128 cases Arch Dermatol 2001 137 1421 1425 11708944 10.1001/archderm.137.11.1421

[B16] Page PZ Page GP Ecosse E Korf BR Leplege A Wolkenstein P Impact of neurofibromatosis 1 on Quality of Life: a cross-sectional study of 176 American cases Am J Med Genet A 2006 140 1893 1898 16906549 10.1002/ajmg.a.31422

[B17] Oostenbrink R Spong K de Goede-Bolder A Landgraf JM Raat H Moll HA Parental reports of health-related quality of life in young children with neurofibromatosis type 1: influence of condition specific determinants J Pediatr 2007 151 182 186 186 e181-182 10.1016/j.jpeds.2007.03.005 17643775

[B18] Jakacki RI Dombi E Potter DM Goldman S Allen JC Pollack IF Widemann BC Phase I trial of pegylated interferon-alpha-2b in young patients with plexiform neurofibromas Neurology 2011 76 265 272 10.1212/WNL.0b013e318207b031 21242495 PMC3034394

[B19] Li F Munchhof AM White HA Mead LE Krier TR Fenoglio A Chen S Wu X Cai S Yang FC Ingram DA Neurofibromin is a novel regulator of RAS-induced signals in primary vascular smooth muscle cells Hum Mol Genet 2006 15 1921 1930 10.1093/hmg/ddl114 16644864

[B20] Guyatt GH Veldhuyzen Van Zanten SJ Feeny DH Patrick DL Measuring quality of life in clinical trials: a taxonomy and review CMAJ 1989 140 1441 1448 2655856 PMC1269981

[B21] Varni JW Seid M Kurtin PS Pediatric health-related quality of life measurement technology: a guide for health care decision makers JCOM 1999 6 33 40

[B22] Wells GA Russell AS Haraoui B Bissonnette R Ware CF Validity of quality of life measurement tools–from generic to disease-specific J Rheumatol Suppl 2011 88 2 6 10.3899/jrheum.110906 22045972

[B23] Craig JB Govender S Neurofibromatosis of the cervical spine. A report of eight cases J Bone Joint Surg Br 1992 74 575 578 1624519 10.1302/0301-620X.74B4.1624519

[B24] Cnossen MH de Goede-Bolder A van den Broek KM Waasdorp CM Oranje AP Stroink H Simonsz HJ van den Ouweland AM Halley DJ Niermeijer MF A prospective 10 year follow up study of patients with neurofibromatosis type 1 Arch Dis Child 1998 78 408 412 10.1136/adc.78.5.408 9659085 PMC1717584

[B25] Drouet A Wolkenstein P Lefaucheur JP Pinson S Combemale P Gherardi RK Brugieres P Salama J Ehre P Decq P Creange A Neurofibromatosis 1-associated neuropathies: a reappraisal Brain 2004 127 1993 2009 10.1093/brain/awh234 15289270

[B26] Till SH Amos RS Neurofibromatosis masquerading as monoarticular juvenile arthritis Br J Rheumatol 1997 36 286 288 10.1093/rheumatology/36.2.286 9133949

[B27] Hersh JH Health supervision for children with neurofibromatosis Pediatrics 2008 121 633 642 10.1542/peds.2007-3364 18310216

[B28] Boulanger JM Larbrisseau A Neurofibromatosis type 1 in a pediatric population: Ste-Justine’s experience Can J Neurol Sci 2005 32 225 231 16018159 10.1017/s0317167100004017

[B29] Khosrotehrani K Bastuji-Garin S Zeller J Revuz J Wolkenstein P Clinical risk factors for mortality in patients with neurofibromatosis 1: a cohort study of 378 patients Arch Dermatol 2003 139 187 191 10.1001/archderm.139.2.187 12588224

[B30] Scalzone M Coccia P Ruggiero A Riccardi R Neurofibromatosis type 1 clinical features and management Pediatr Med Chir 2009 31 246 251 20333883

[B31] Lynch TM Gutmann DH Neurofibromatosis 1 Neurol Clin 2002 20 841 865 10.1016/S0733-8619(01)00019-6 12432832

[B32] Ferner RE The neurofibromatoses Pract Neurol 2010 10 82 93 10.1136/jnnp.2010.206532 20308235

[B33] Ferner RE Huson SM Thomas N Moss C Willshaw H Evans DG Upadhyaya M Towers R Gleeson M Steiger C Kirby A Guidelines for the diagnosis and management of individuals with neurofibromatosis 1 J Med Genet 2007 44 81 88 10.1136/jmg.2007.049122 17105749 PMC2598063

[B34] Huson SM Acosta MT Belzberg AJ Bernards A Chernoff J Cichowski K Gareth Evans D Ferner RE Giovannini M Korf BR Back to the future: proceedings from the 2010 NF Conference Am J Med Genet A 2011 155A 307 321 21271647 10.1002/ajmg.a.33804 PMC3079924

[B35] PedsQL™ (Pediatric Quality of Life Inventory) http://www.mapi-trust.org/services/questionnairelicensing/catalog-questionnaires/84-pedsql

[B36] Varni JW Sherman SA Burwinkle TM Dickinson PE Dixon P The PedsQL family impact module: preliminary reliability and validity Health Qual Life Outcomes 2004 2 55 10.1186/1477-7525-2-55 15450120 PMC521692

[B37] Varni JW Seid M Smith Knight T Burwinkle T Brown J Szer IS The PedsQL in pediatric rheumatology: reliability, validity, and responsiveness of the Pediatric Quality of Life Inventory Generic Core Scales and Rheumatology Module Arthritis Rheum 2002 46 714 772 10.1002/art.10095 11920407

[B38] Varni JW Burwinkle TM Katz ER Meeske K Dickinson P The PedsQL in pediatric cancer: reliability and validity of the pediatric quality of life inventory generic core scales, multidimensional fatigue scale, and cancer module Cancer 2002 94 2090 2106 10.1002/cncr.10428 11932914

[B39] Varni JW Burwinkle TM Berrin SJ Sherman SA Artavia K Malcarne VL Chambers HG The PedsQL in pediatric cerebral palsy: reliability, validity, and sensitivity of the generic core scales and cerebral palsy module Dev Med Child Neurol 2006 48 442 449 10.1017/S001216220600096X 16700934

[B40] Smith JA Flowers P Larkin m Interpretative Phenomenological Analysis: Theory, Method and Research 2009 London: Sage Publications

[B41] Pringle J Drummond J McLafferty E Hendry C Interpretative phenomenological analysis: a discussion and critique Nurse Res 2011 18 20 24 21560922 10.7748/nr2011.04.18.3.20.c8459

[B42] Iannaccone ST Hynan LS Morton A Buchanan R Limbers CA Varni JW The PedsQL in pediatric patients with spinal muscular atrophy: feasibility, reliability, and validity of the pediatric quality of life inventory generic core scales and neuromuscular module Neuromuscul Disord 2009 19 805 812 10.1016/j.nmd.2009.09.009 19846309 PMC2796341

[B43] Davis SE Hynan LS Limbers CA Andersen CM Greene MC Varni JW Iannaccone ST The PedsQL in pediatric patients with duchenne muscular dystrophy: feasibility, reliability, and validity of the pediatric quality of life inventory neuromuscular module and generic core scales J Clin Neuromuscul Dis 2010 11 97 109 10.1097/CND.0b013e3181c5053b 20215981

[B44] McHorney CA Ware JE Jr Lu JF Sherbourne CD The MOS 36-item Short-Form Health Survey (SF-36): III. Tests of data quality, scaling assumptions, and reliability across diverse patient groups Med Care 1994 32 40 66 10.1097/00005650-199401000-00004 8277801

[B45] Cronbach L Coefficient alpha and the internal structure of tests Pyschometrika 1951 16 297 334 10.1007/BF02310555

[B46] Nunnally JC Bernstien IH Psychometric theory 1994 3 New York: McGraw HIll

[B47] Pedhazur EJ Schmelkin LP Measurement, Design, and Analysis: An Integrated Approach 1991 Hilsdale, NJ: Erlbaum

[B48] Hays RD Anderson R Revicki D Psychometric considerations in evaluating health-related quality of life measures Qual Life Res 1993 2 441 449 10.1007/BF00422218 8161978

[B49] Varni JW Seid M Kurtin PS PedsQL 4.0: reliability and validity of the Pediatric Quality of Life Inventory version 4.0 generic core scales in healthy and patient populations Med Care 2001 39 800 812 10.1097/00005650-200108000-00006 11468499

[B50] McHorney CA Ware JE Jr Rogers W Raczek AE Lu JF The validity and relative precision of MOS short- and long-form health status scales and Dartmouth COOP charts. Results from the Medical Outcomes Study Med Care 1992 30 MS253 MS265 10.1097/00005650-199205001-00025 1583937

[B51] Weissberg-Benchell J Zielinski TE Rodgers S Greenley RN Askenazi D Goldstein SL Fredericks EM McDiarmid S Williams L Limbers CA Pediatric health-related quality of life: feasibility, reliability and validity of the PedsQL transplant module Am J Transplant 2010 10 1677 1685 10.1111/j.1600-6143.2010.03149.x 20642689

[B52] Ozonoff S Cognitive impairment in neurofibromatosis type 1 Am J Med Genet 1999 89 45 52 10.1002/(SICI)1096-8628(19990326)89:1<45::AID-AJMG9>3.0.CO;2-J 10469436

